# Factors associated with late presentation to HIV/AIDS care in South Wollo ZoneEthiopia: a case-control study

**DOI:** 10.1186/1742-6405-8-8

**Published:** 2011-02-28

**Authors:** Yeshewas Abaynew, Amare Deribew, Kebede Deribe

**Affiliations:** 1 Dessie Health Science College, Dessie, Ethiopia; 2 Department of Epidemiology, Jimma University, Jimma, Ethiopia; 3 American Refuge Committee International, South Darfur, Nyala, Sudan

## Abstract

**Background:**

Access to free antiretroviral therapy in Sub-Saharan Africa has been steadily increasing. The success of large-scale antiretroviral therapy programs depends on early initiation of HIV/AIDs care. The purpose of the study was to examine factors associated with late presentation to HIV/AIDS care.

**Methods:**

A case-control study was conducted in Dessie referral and Borumeda district hospitals from March 1 to 31, 2010, northern Ethiopia. A total of 320 study participants (160 cases and 160 controls) were included in the study. Cases were people living with HIV/AIDS (PLHA) who had a WHO clinical stage of III or IV or a CD4 lymphocyte count of less than 200/uL at the time of the first presentation to antiretroviral treatment (ART) clinics. Controls were PLHA who had WHO stage I or II or a CD4 lymphocyte count of 200/uL or more irrespective of clinical staging at the time of first presentation to the ART clinics of the hospitals cases and controls were interviewed by trained nurses using a pre-tested and structured questionnaire. In-depth interviews were conducted with ten health workers and eight PLHA.

**Results:**

PLHA who live with their families [OR = 3.29, 95%CI: 1.28-8.45)], lived in a rented house [OR = 2.52, 95%CI: 1.09-5.79], non-pregnant women [OR = 9.3, 95% CI: 1.93-44.82], who perceived ART have many side effects [OR = 6.23, 95%CI:1.63,23.82)], who perceived HIV as stigmatizing disease [OR = 3.1, 95% CI: 1.09-8.76], who tested with sickness/symptoms [OR = 2.62, 95% CI: 1.26-5.44], who did not disclose their HIV status for their partner [OR = 2.78, 95% CI: 1.02-7.56], frequent alcohol users [OR = 3.55, 95% CI: 1.63-7.71] and who spent more than 120 months with partner at HIV diagnosis[OR = 5.86, 95% CI: 1.35-25.41] were significantly associated with late presentation to HIV/AIDS care. The qualitative finding revealed low awareness, non-disclosure, perceived ART side effects and HIV stigma were the major barriers for late presentation to HIV/AIDS care.

**Conclusions:**

Efforts to increase early initiation of HIV/AIDS care should focus on addressing patient's concerns such as stigma, drug side effects and disclosure.

## Introduction

Sub-Saharan Africa remains the most affected region in the global AIDS epidemic; with an estimated 22.5 million people living with HIV [1]. The health status of HIV positive individuals at the time of antiretroviral (ART) initiation plays a crucial role in the success of treatment [2-6]. HIV positive individuals with advanced HIV disease at the time of ART initiation are less likely to respond to treatment, are more likely to pose financial strain on health services, and have a higher mortality rate compared to those who initiate earlier [2-4]. In addition, late presentation poses a higher cumulative risk of HIV transmission to others, considering that earlier presentation and HIV-suppressing treatment might otherwise reduce viral load and risk of transmission[5]. A large proportion of HIV-infected individuals in the developed world, roughly 15%-43%, present at clinics for care with advanced or severe disease (WHO stage 3 or 4 or CD4 lymphocyte count ≤ 200 cells/uL) [7].

The introduction of ART has offered hope to people living with HIV/AIDS and has been credited with improving the quality of life significantly and reducing mortality. However, access to ART remains limited, especially in developing countries, in Sub-Saharan Africa [8].

Late presentation prevents people living with HIV/AIDS from obtaining the maximal benefit of being screened for tuberculosis and sexually transmitted infections, receiving timely antiretroviral therapy, and benefiting from educational and prophylactic interventions that are more effective when implemented earlier and that can prevent further infections [5]. HIV infection without antiretroviral therapy in the vast majority of infected individuals progressively destroys the immune system leading to opportunistic diseases and death [9,10].

The reason for late presentation is not clearly studied in Ethiopia. There are multiple factors associated with delay to seek care and treatment, including delay in HIV testing and delay in accessing care once HIV status is known. Exploring the barriers of early HIV/AIDS care will help decision makers to improve the access to HIV treatment. The purpose of this study was to explore the demographic, behavioral and clinical barriers to HIV/AIDS care.

## Methods

A case control study was conducted from March 1-31, 2010 in Dessie referral hospital and Borumeda district hospital in south Wollo Zone, north-east Ethiopia. South Wollo is one of the 11 zones of Amhara regional state. The zone is found in the north-east part of Ethiopia. In the hospitals, voluntary counseling and testing (VCT), prevention of mother to child transmission (PMTCT), ART and treatment of opportunistic infection services are available. The study population consisted of sampled cases and controls who had visited HIV/AIDS care in the ART clinic of the hospitals during data collection period. Cases were HIV positive individuals who had WHO clinical stage III or IV irrespective of CD4 lymphocyte count or a CD4 lymphocyte count of less than 200/uL irrespective of clinical staging at the time of first presentation to the ART clinics of the hospitals. Controls were HIV positive individuals who had WHO stage I or II or a CD4 lymphocyte count of 200/uL or more irrespective of clinical staging at the time of first presentation to the ART clinics of the hospitals.

The sample size was calculated using Epi info version 3.3.2.0 by considering the following assumptions: the proportion of literate individuals among the controls 58% [8], 95% CI, 80% power and case to control ratio of 1:1 to detect an odds ratio of 2.0. The total sample size was 320(160 cases and 160 controls). Previous studies have indicated that educated individual's present themselves early for ART. Educational status was chosen as an independent variable since it gave maximum sample size.

HIV positive individuals were classified into two groups based on their CD4 lymphocyte count and WHO clinical HIV staging at the time of first presentation to the ART clinics of the hospitals. Source population for cases and controls were identified by reviewing the initial clinic visit medical records at the ART clinics. From the source population of cases and controls, the study populations (160 cases and 160 controls) were randomly selected using simple random sampling technique. Unique patients' identification numbers in the ART clinics were used as a sampling frame for the simple random selection process. HIV positive individuals less than 15 years of age and who received prior HIV/AIDS care were excluded from the study. The selection of cases and control in the study hospitals is described in Table 1. The data were collected by trained ART case managers and ART nurses using an interviewer-administered, pre-coded and pre-tested structured questionnaire to address the necessary information. The questionnaire was developed in English after reviewing relevant literatures and it was translated into Amharic (local language). To check for its consistency, the questionnaire was back translated into English by other people who have the experience of similar work. The contents of the questionnaires included socio-demographic characteristics, HIV testing and behavioral factors such as use of alcohol, knowledge, belief and attitude towards HIV/AIDs and health system related factors. Attitudes and beliefs about ART and their relationship with late presentation to HIV/AIDS care were examined using 11 items. Based on these questions, an answer consistent with negative attitude towards HIV was scored with one point. An answer not consistent with negative attitude towards HIV was scored as zero points. A total attitude score for HIV was created by summing the scores of the 11 questions. The attitude score ranged from 0 to 11, with the higher the score, the greater the degree of attitude towards HIV. Individuals who had an attitude score of equal to or greater than the mean score of the study population were categorized as having negative attitude towards HIV/AIDS. Perceived stigma was assessed using 5 items in the same way as the attitude scores.

**Table 1:** Factors independently associated with late presentation to HIV/AIDS Care in public hospitals of South Wollo, April 2010.

Variables	Cases N (%)	Controls N (%)	Crude OR (95%CI)	Adjusted OR (95%CI)
**Living arrangements**				
Living alone	34 (21.2)	26 (16.2)	1	1
Families	94 (58.8)	69 (43.1)	1.0(0.57-1.894)	3.3(1.279-8.447)
Husband/wife	29 (18.1)	60 (37.5)	0.4(0.188-0.73)	0.6(0.173-2.424)
Others	3 (1.9)	5(3.1)	0.5(0.10-2.097)	0.7(0.046-10.63)
**Ownership residence**				
Owning	62(38.8)	100(62.5)	1	1
Renting	98(61.2)	60(37.5)	2.6(1.678-4.14)	2.5(1.093-5.793)
**Pregnancy status**				
Yes	7(6.6)	17(19.1)	1	1
No	99(93.4)	72(80.9)	3.3(1.316-8.47)	9.3(1.928-44.82)
**Perceived ART side effects**				
Yes	31(19.4)	18(11.2)	5.6(2.46-13.03)	6.2(1.63-23.82)
No	115(71.9)	96(60)	3.9(2.041-7.59)	2.3(0.778-6.568)
I don't know	14(8.8)	46(28.8)	1	1
**Perceived HIV stigma**				
high	36(22.5)	15(9.4)	2.8(1.47-5.367)	3.1(1.094-8.764)
low	124(77.5)	145(90.6)	1	1
**Symptoms at HIV testing**				
Yes	75(46.9)	53(33.1)	1.8(1.13-2.80)	2.6(1.258-5.437)
No	85(53.1)	107(66.9)	1	1
**HIV status disclosure to partner**				
Yes	29(18.1)	61(38.1)	1	1
No	74(46.2)	47(29.4)	3.3(1.866-5.88)	2.8(1.023-7.56)
Not disclosed for others	57(35.6)	52(32.5)	2.3(1.29-4.12)	3.7(1.30-10.32)
**Ever alcohol use**				
Never	87(54.4)	117(73.1)	1	1
Some times	19(11.9)	19(11.9)	1.4(0.67-2.69)	0.9(0.380-2.43)
Most of the times	54(33.8)	24(15.0)	3.03(1.74-5.27)	3.6(1.63-7.71)
**Time spent with steady partner**				
Less than 24 month	8(25.8)	21(21.9)	1	1
25 to 120 months	23(32.4)	36(37.5)	1.7(0.64-4.415)	2.9(0.65-13.15)
Greater than 120 months	40(41.8)	39(40.6)	2.7(1.07-6.80)	5.9(1.35-25.41)

To triangulate the findings of the case-case control study, in-depth interviews with health workers engaged in HIV/AIDS care and selected cases and controls were done. Data were cleaned for inconsistencies and missing value and analyzed using SPSS version 16.0-statistical software. Descriptive statistics were done to the socio-demographic characteristics of the study participants. To assess the association between the different barriers of early HIV/AIDS care (independent variables) with the dependent variable, first bivariate analysis was done to control for the effect of confounding factors, stepwise multiple logistic regression was done. All independent variables with p-value less than 0.05 in the bivariate analyses were included in the final multiple logistic regression model.

The qualitative data were transcribed and analyzed using thematic areas.

Ethical clearance was obtained from the Institutional Review Committee of Jimma University. Written consent was obtained from the study participants.

## Results

A total of 320 HIV positive individuals (160 cases and 160 controls) participated in the study. The reliability (Cronbatch's **α**) of the knowledge, attitude and beliefs items was 0.9. Attitudes and beliefs of people living with HIV/AIDS were not associated with late presentation to HIV/AIDS care (P > 0.05).

Variables which showed association with late presentation to HIV/AIDS care in the bivariate analysis such as occupational status at HIV diagnosis, area of residence, marital status, living arrangements, ownership of residence, pregnancy status, understanding all HIV positive are eligible to HIV/AIDS care, perceiving ART have many side effects, HIV stigma, awareness of VCT, source of information about VCT, knowing where to get VCT, HIV testing with symptoms/sickness, HIV testing with medical consultation, HIV status disclosure to partner, HIV status disclosure to families, ever alcohol use, alcohol use in the previous year, having steady partner at HIV diagnosis, time spent with steady partner, health-seeking behaviour when felt at risk, prior experience of health system and travel time to hospital were further evaluated in the multivariable model. Finally, living arrangement, ownership residence, pregnancy status, perceived ART side effects, perceived HIV stigma, symptoms at HIV diagnosis, HIV status disclosure to partner, ever alcohol use and time spent with steady partner were found to be independent factors of late presentation to HIV/AIDS care (Table 1).

In multivariable analysis, HIV positive individuals who live with families were 3.29 times more likely to present late to HIV/AIDS care than HIV positive individuals who live alone [OR = 3.29, 95%CI: 1.28-8.45)]. HIV positive individuals who live with renting house were 2.52 times more likely to present late to HIV/AIDS care than HIV positive individuals who live with owning house [OR = 2.52, 95%CI: 1.09-5.79]. Non-pregnant women were 9.3 times more likely to present late to HIV/AIDS care than pregnant women [OR = 9.3, 95% CI: 1.93-44.82]. HIV positive individuals who perceived ART have many side effects were 6.23 time more likely to present late to HIV/AIDS care than HIV positive individuals who did not know about side effects of ART drugs [OR = 6.23, 95%CI: 1.63-23.82)]. People living with HIV/AIDS who perceived HIV stigma were 3.1 times more likely to present late to HIV/AIDS care than those who did not perceive HIV stigma [OR = 3.1, 95% CI: 1.09-8.76]. HIV positive individuals tested with sickness/symptoms were 2.62 times more likely to present late to HIV/AIDS care than those tested without HIV related symptoms at first HIV diagnosis [OR = 2.62, 95% CI: 1.26-5.44)]. HIV positive individuals who did not disclose their HIV status for their partners were 2.78 times more likely to present late to HIV/AIDS care than those disclosed their HIV status for their partners [OR = 2.78, 95% CI: 1.02, 7.56].

HIV-infected individuals who were frequent alcohol drinkers were 3.55 more likely to present late to HIV/AIDS care than non-alcohol user HIV positive individuals [OR = 3.55, 95% CI: 1.63-7.71]. HIV positive individuals who spent more than 120 months with partners at HIV diagnosis[OR = 5.86, 95% CI: 1.35-25.41)] were 5.86 times more likely to present late to HIV/AIDS care than those spent less than 24 months (Table 1).

The qualitative finding revealed that low level awareness/inadequate knowledge about HIV/AIDS, HIV testing and HIV/AIDS care, perceived HIV stigma, perceived side effects of ART drugs, inadequacy of social support, inadequate coverage of health education provided to the community, low participation of the community, unavailability of transportation to ART clinic/VCT center, and substance use were the barriers faced by people living with HIV/AIDS for early presentation to HIV/AIDS care as perceived by interviewed health workers and HIV positive individuals(Table 2).

**Table 2:** Examples of interview extracts of HIV positive individuals and health workers concerning barriers for HIV/AIDS care, South Wollo, April 2010.

Barriers for HIV/AIDS care	HIV positive individuals and health workers citations
Inadequate knowledge about HIV/AIDS and HIV/AIDS care	*''...I have inadequate knowledge about HIV/AIDS and HIV care .I came to the health facility when I was seriously sick''*
	***(HIV-positive individual (Case) from Borumeda Hospital)***
	
Fear of side effects	*"...The side effects of almost all antiviral drugs are the most difficulty faced by people living with HIV/AIDS to present early for HIV/AIDS care."*
	***(Pharmacy technician from Dessie Referral Hospital)***
	
Perceived HIV stigma	*''.........Some people living with HIV/AIDS did not disclose their HIV status for others because HIV positive individuals experienced HIV stigma and discrimination, losing jobs, breaking relationships and disturbance of families.''*
	***(PMTCT nurse from Borumeda Hospital)***
	
Non-disclosure of HIV status	*'HIV positive individuals were highly stigmatized by the community. I didn't want to disclose my HIV status to others.''*
	***HIV-positive individual (Case) from Dessie Referral Hospital)***

## Discussion

This study has investigated factors that are correlated with late presentation to HIV/AIDS care for the first time in Ethiopia. PLHA who live with their families, lived in a rented house, non-pregnant women, who perceived ART have many side effects, who perceived HIV as stigmatizing disease, who tested with sickness/symptoms, who did not disclose their HIV status for their partner, frequent alcohol drinkers and who spent more than 120 months with partner at HIV diagnosis were significantly associated with late presentation to HIV/AIDS care. The findings and the implications of the study were discussed in light with previous studies.

Non-pregnant women at HIV diagnosis were significantly associated with late presentation to HIV/AIDS care which is consistent with the studies done in Uganda [8], and Thailand [11]. The lower likelihood of pregnant women presenting late to HIV/AIDS care could be explained by the current programs to routinely offer HIV testing and treatment for the prevention of mother-to-child transmission in antenatal clinics are successfully linking most HIV-infected women with HIV/AIDS care.

HIV positive individuals who did not disclose their HIV status to their spouses/partners were more likely to present late to HIV/AIDS care compared with those who disclosed their HIV status, studies done elsewhere [9,12] supports this finding. This could be explained that the desire to hide one's HIV-positive status from a spouse may inhibit HIV care-seeking. The qualitative finding also revealed that non-disclosure of HIV status was associated with late presentation to HIV/AIDS care.

HIV positive individuals who consume alcohol were significantly associated with late presentation to HIV/AIDS care which is consistent with the finding of studies done India [13] and German KompNet Cohort [14] where alcohol consumption has been shown to be related to not receiving treatment. This suggests that there is lack of readiness for behavior change among people living with HIV/AIDS to start the HIV/AIDS care early.

HIV positive individuals who experienced HIV stigma were significantly associated with late presentation to HIV/AIDS care. Similarly studies done in India [13], Mozambique [15] and Zambia [16], reported that a family member did not want them to take ARVs. Other study [17] found that ''feeling well'' was associated with lower rates of care linkage after diagnosis. This could be explained that AIDS stigma affects preventive behaviors such as HIV test-seeking behavior and care-seeking behavior upon diagnosis even as access to care has become more common.

HIV positive individuals who have symptoms/sickness at HIV diagnosis were significantly associated with late presentation to HIV/AIDS care which is consistent with the finding of the studies done elsewhere [16,18]. This could be partly explained by that people living with HIV/AIDS presented late in the course of their disease or more likely to be diagnosed at advanced stages of disease progression.

HIV positive individuals who perceived ART have side effects were significantly associated with late presentation to HIV/AIDS care which is similar to the finding of the studies done in India [8], Zambia [16] and Cameroon [19], showed that perceiving ART as a therapy associated with side effects. This finding indicated that a lack of information about HIV/AIDS care impeded their initiation of medical care the necessity to seek medical care and the benefits of early medical interventions.

HIV positive individuals who are in long term relationship with their partner at HIV diagnosis were more likely to present late to HIV/AIDS care than their counterparts which is similar to a study done in Venezuela [20] that showed there was an increased trend to present late the longer a person had a steady partner. Other study [21] showed that late diagnosis is more frequent among people with children in a longstanding steady partnership. This could be explained related to fear of outcomes of HIV sero-status disclosure to long time partner or it might be explained with individuals in longer partnerships were more likely to perceive they were at no risk and may not consider themselves at risk and thus do not seek voluntary counseling and testing, which leads to progression of the disease.

The combination of methods as well as the involvement of people living with HIV/AIDS and health workers allowed a cross-validation of data and possibly a minimization of biases. Using case-control study design for assessing factors associated with late presentation to HIV/AIDS care was considered as the good side of the study.

The study had some limitations that could have influenced the findings. The study relies on participants' self report of historical events (recall biases could have been present). The analyses of late presentation would not represent the characteristics of HIV positives who never attended ART clinic and who attended in the health centers (selection bias). Moreover, the use of ART nurses and ART case managers in the hospitals as data collectors might have introduced an interviewer bias and social desirability bias. Finally the instrument used in this study is not generic which was not validated in Ethiopia.

## Conclusion

In conclusion, non-pregnant women, those who live with families, who live in renting house, those who consume alcohol, non-disclosure of HIV status to partner, who perceive HIV stigma, those who have symptoms/sickness at HIV diagnosis, who perceived ART have side effects and long standing couples were associated with late presentation to HIV/AIDS care. To improve presentation to HIV/AIDS care interventions, whether designed to promote HIV testing or early entry into care, should target non-pregnant women, those who live with families, those who live in renting houses, those consume alcohol, who perceived HIV stigma, those who have symptoms at HIV diagnosis and those perceived ART have side effects. In addition the HIV testing programs may help accelerate initiation of HIV care by encouraging HIV serostatus disclosure to partners on positive diagnosis.

## Competing interests

The authors declare that they have no competing interests.

## Authors' contributions

YA, AD conceived the study designed the study, analyzed the data and wrote the first draft. KD analyzed the data and contributed to the draft manuscript. All authors contributed to the manuscript and approved its final version.
